# Painful Dream of Motherhood: A Hermeneutic Phenomenological Study of Married Women with Ectopic Pregnancy in the Islamic Republic of Iran

**DOI:** 10.1007/s10943-023-01936-y

**Published:** 2023-11-25

**Authors:** Seyed Ahmad Firouzabadi, Setareh Sarshad Shadman, Parvaneh Rezasoltani, AbouAli Vedadhir

**Affiliations:** 1 Department of Development Studies, Faculty of Social Sciences, University of Tehran, Tehran, 14117-13118 Iran; 2 Department of Midwifery and Reproductive Health, School of Nursing & Midwifery, Reproductive Medical Science, Guilan, Iran; 3 Department of Anthropology, Faculty Social Science, Beside Shariati Hospital, University of Tehran, Jalale Aleh Ahmad Highway, P.O. Box: 1411713118, Tehran, Iran; 4 Population Health Sciences, University of Bristol, Canynge Hall, Bristol, BS8 2PS UK

**Keywords:** Ectopic pregnancy, Hermeneutic/interpretative phenomenological analysis, Islamic sharia, Trauma

## Abstract

Ectopic pregnancy (EP) is a significant cause of maternal morbidity and mortality. This study aimed to explore the understanding and experience of women with EP in the Islamic Republic of Iran. This qualitative study carried out through a Heideggerian hermeneutic/interpretative phenomenological approach, using face-to-face semi-structured phenomenological interviews with twenty-five participants referred to a public maternity hospital in Rasht, Iran. Data were collected and analyzed using the seven-step analytical approach of Dickelman et al*.* (The NLN criteria of appraisal of baccalaureate programs: A critical hermeneutic analysis, NLN Press, 1989; Journal of Nursing Education. 32:245–250, 1993) to phenomenological studies. The results reveal how living in the shadow of Islamic Sharia Law in Iran turns EP into a trauma and creates a different experience and meaning of EP for each woman. In this view, multiple factors, including ‘family support’ and ‘faith in Islamic Sharia,’ have determined how married women experience sociocultural and psychological consequences of EP. These findings apply to women with EP in Iran. Given that EP is more than an anomalous pregnancy with socially culturally constructed suffering in the context of the Islamic Republic of Iran. Hence, policymakers and healthcare providers should consider a multidimensional approach to this devastating event in pregnancy and support and empower the women whose dream of motherhood is jeopardized and terminated by the experience of EP.

## Introduction

Ectopic pregnancy (EP) is the most common cause of female mortality in the first trimester of gestation that often requires runnable surgical intervention immediately (Lawani et al., 2013). In another word, any pregnancy implanted outside the endometrial cavity is defined as an EP (Jurkovic & Salman, 2016).

About 2% of all pregnancies are complicated by and termed EP (Brüggmann et al., 2017). According to the World Health Organization (WHO), high-risk pregnancies, including EP, account for nearly 75 percent of all maternal deaths and could lead to infertility and sterility in future (WHO, 2023).

In addition to the physiological and psychological threats caused by EP, in some cultures, any failure or loss in pregnancy and childbirth could be stigmatized, particularly in the pronatalist Islamic World, where women are fully expected to have children (Chaudhry, 2010).

In Iran, after the 1979 Islamic Republic Revolution, girls are justifiably allowed to get married from the age of nine (Khomeini, 2014, p. 241), the age at which they can engage in intimate marital relations, not at the age of menstruation. Likewise, according to Iranian Family Laws, if a woman is infertile or unable to give birth, her husband will be allowed to divorce her (Mir-Hosseini, 1993).

For women, living in the shadow of Islamic Sharia Law in post-revolutionary Iran means that being a wife entails having a successful pregnancy and childbirth, at least once, as becoming a mother is unquestionably a central part of women’s identity in the pronatalist societies.

Hence, from a phenomenological perspective, EP is a dynamic and multidimensional issue that can be understood and experienced differently worldwide. In this view, EP in Iran can be considered a complex sociocultural phenomenon that could influence a couple’s marital life and trigger a constant identity crisis for women.

A brief review of the previous literature suggests that few studies have been conducted about EP specifically using qualitative research methods worldwide. For instance, Yuk et al. (2013) analyzed Korean National Health Insurance data from January to December 2009 to identify whether socioeconomic status (SES) in association with age contributes to the incidence of EP. They found that older age and low SES are risk factors for EP in the Republic of Korea.

Foster et al. (2011) undertook a national qualitative study to explore the relationship between the Ethical and Religious Directives for Catholic Health Care Services (*the Directives*), hospital policies regarding EP management, and clinical practices. They recruited participants at non-Catholic, longstanding Catholic, and recently merged facilities and conducted focused interviews with 24 physicians at 16 hospitals in 10 states.

The results of this study revealed that some interpretations of *the Directives* as the dominant provider of care in Catholic-affiliated hospitals and the head force in prohibiting the provision of abortion in almost all circumstances in the United States are precluding physicians from providing women with EP with information about and access to a full range of treatment options and are resulting in practices that delay care and may expose women to unnecessary risks.

A qualitative study by Spillane et al. (2018) was also performed and conducted to gain insight into Irish women’s experience of EP. They purposefully recruited and phenomenological interviewed seven participants who had experienced an EP in a large tertiary-level Irish maternity hospital. The results showed that lack of bereavement counseling and satisfactory completion of outpatient care hinder closure and recovery for women who experienced EP in Ireland.

This study has key insights and implications for the health care of women who experience an EP principally, concerning how they are managed from diagnosis to completion of treatment.

Notwithstanding the above-limited studies, no study found how Iranian married women understand and experience EP in the shadow of Islamic Sharia Law in the post-revolutionary era.

Hence, this study aimed to explore how Iranian married women experience EP and its specific sociocultural and mental consequences before and after the treatment. More specifically, the main objective of this study was to address the following research question: *“How does it look like to experience EP for married women living in the context of existing Islamic Sharia Law in Iran?”.*

## Methodology

### Design

This study tends to explore perception and lived experience of participants and to construct knowledge through the conceptualization of their experiences and meanings of EP by taking a qualitative research approach. The principles of Heideggerian hermeneutic/interpretative phenomenology were used for designing, gathering, and handling data and pieces of evidence in this study, as it promotes a better and more comprehensive understanding of Iranian married women meanings and experiences with EP.

This interpretative approach in qualitative research is an appropriate ‘fit’ for those researchers seeking a deeper understanding of phenomenon (Miles et al., 2013) and is committed to the investigation of how people perceive and make sense of their life experience. It is phenomenological by nature as it is concerned with exploring experience in its terms (Smith et al., 2009).

### Participants and Setting

Twenty-five married women who had experienced an EP and referred to a public maternity hospital in Rasht City of Iran were recruited purposively and interviewed phenomenologically. The sampling procedure was ended with maximum variation from April 2017 to April 2018, and participated women were in different age groups and received various treatment methods. In addition, those married women misdiagnosed with EP excluded. There are three type of treatment, regarding EP:

1. *Expectant treatment:* controlling patient’s biological reactions during EP, which is expected to resolve naturally without any intervention. The time period for this method varies from three to seven days.
2. *Medical treatment:* After one week and the expectant treatment failure, methotrexate injection is given in three divided doses on every alternative day, at least for one week. Methotrexate injection may cause some side effects, namely dizziness, drowsiness, headache, swollen and tender gums, decreased appetite, reddened eyes and hair loss. Besides, due to these side effects, patients are not allowed to get pregnant for six months. Nevertheless, these disadvantages outweigh the surgery expenses, both for patients and the government.
3. *Surgical treatment:* The candidates for *surgical treatment* include women who are not suitable to or have failed methotrexate *treatment. Surgery can be laparotomy or laparoscopy, depending on the conditions and facilities available. Although laparoscopy has less disadvantages, not all patients are lucky enough to be candidate for this method, due to facility limitations. Owing to severe bleeding they often lose a* fallopian tube or uterus which means impaired fertility in future. (The National Health Service/NHS, 2018)

Throughout the recruitment process, the participants were well informed that the study was designed to explore how they experience EP through their own meanings and narratives. Also, they were given vital information regarding the research ethical considerations including that participating in this research is entirely voluntary.

Overall, more than two-thirds of the married women belonged to the 25–34 age group (68%), and the rest were between 15–24 and 35–44 age groups. Twelve women graduated from secondary school, and just eight women were higher educated. The women’s SES was measured by three components of education, income, and occupation.

As can be viewed in Table 1, most women were classified as low socio-economic status in terms of SES. About half of the women had at least one child. As the result of recruiting the participants from all three treatment groups, the experience of women with different EP treatments and consequences was considered in the study. Socio-demographic details of the participating women are described in Table 1.

**Table 1:** Socio-demographic overview of the participants (*n* = 25)

Characteristics		Frequency (%)
Age	15–24	4 (16)
	25–34	17 (68)
	35–44	4 (16)
Education	Primary	5 (20)
	Secondary/Diploma	12 (48)
	University	8 (32)
Socio-Economic Status (SES)	High	2 (8)
	Middle	9 (36)
	Low	14 (56)
Number of Children	One or more	12 (48)
	Childless	13 (52)
*Treatment Outcome*	No Complications	8 (32)
	Medicine Side effects	8 (32)
	Impaired fertility	9 (36)

### Data Collection

In this study, the technique of data collection was the phenomenological interview. Interviews were led by a team member of the study (second author) with over ten years’ experience in researching reproductive loss, abortion and infertility treatments in the Islamic Republic of Iran.

Following three pilot interviews, in order to be familiar with the field of research and make a better connection with typical patients of EP, the data were collected using semi-structured, in-depth and face-to-face phenomenological interviews with twenty-five participants. The interviews lasted between 30 min and an hour each (Sum: 1195 min & Average: 45 min) and were conducted at any time and places they preferred (for instance, a private booked room at the hospital, a café, the participants’ workplace, and even their house).

Their contribution to this study was about two in-depth face-to-face phenomenological interviews. The first interview was performed in the hospital during the session of treatment process (7–40 days), and the second one carried out about six months later, when they had been discharged of the hospital. These women talked openly about their emotions, feelings, events, hopes and all motivations they experienced during the EP diagnosis and treatment completion process.

At the beginning of the first interview, they were asked the guiding question, *‘What does it look like to have an EP?’* Further probing questions were used to clarify participants’ descriptions and to attain additional evidence during interviews. After six months, the second interview started with new questions; such as *‘What does life look like after an EP?’* and ‘*How do you feel about your life now?’.*

All interviews were digitally recorded and transcribed verbatim in Persian and analyzed consecutively. Throughout all interviews and data collection process, verbal and non-verbal reactions of participants were also observed and considered. The interviewed women were also reminded of their right to withdraw from the study any time. Finally, this cooperation had no known financial, physical, and psychological costs for participants.

### Data Analysis

Data were managed and analyzed using the seven-step analytical approach of Dickelman et al., (1989, 1993). It is a sequential and iterative process that follows Heidegger’s hermeneutic phenomenology (philosophical hermeneutics) and involves the following steps:

- After each interview, the recorded audio files, the field notes and memoing on important cases (such as the participants’ feelings or their other reactions or non-verbal practices) were transcribed and typed word by word. The transcripts were then reviewed several times to get a general understanding of them (*Step 1*).
- For each of the transcripts, an interpretive summary was written to understand and extract meanings hidden in the interviews (*Step* 2).
- The main researcher and other members of the research team reviewed the transcripts to extract the cods and themes. Explicit and implicit meanings were extracted from the manuscripts. These meanings also reflected the context of the interviews and how the participants answered the questions (*Step 3*).
- To clarify and eliminate any disagreement and inconsistencies in the interpretations, the researcher repeatedly returned to the transcripts and sometimes referred to the participants—the hermeneutic cycle (*Step 4*).
- The interpretive summaries were merged into a more general interpretation so that the resulting themes could be related to each other in the best possible way (*Step 5*).
- A final interpretation or structural statement reflecting the connections between the extracted subthemes and themes was prepared— the highest level of hermeneutic analysis (*Step 6*).
- Finally, a draft copy of the extracted themes and subthemes and an excerpt of interview transcripts were provided to the members of the research team and an external reviewer who was familiar with qualitative research, and their comments and suggestions were applied in the final version of the project report (*Step 7*).

All transcripts in this study were translated from Persian to English by the authors and edited and validated by one of senior authors of the team who is fluent in both English and Persian. In sum, iteratively interlocked phases of data collection and analysis took place between April 2017 and April 2018.

### Ethical considerations

Ethical approval for the study was granted by the Ethical Committee of the University of Tehran on 6th March 2017 (*Ref: IR.UT.Rec.1395003*) and relevant authorities of the hospital in Rasht City of Iran. The participants filled in the informed consent forms, so that researchers could be allowed to audio record the interviews.

The confidentiality of information was guaranteed as the name and personal information of interviewees was not mentioned in recordings and transcripts. All recordings, transcripts, and information sheets were given special codes and were kept separately to protect their anonymity.

## Results

Over one hundred initial codes were extracted from the interviews. These codes were integrated and reduced gradually by removing overlapped codes. This iterative analytical process finally identified two master/main themes: ‘Constant struggle’ and ‘Renascence in life.’

These main themes consisted of five themes and seven subthemes. In order to maintain confidentiality, the married women were assigned pseudonyms. Direct quotations from the interviewed married women are employed to represent all main themes, themes, and subthemes.

### Constant Struggle

The circumstances experienced by EP patients were not the same for everyone. By the time, some participants were discharged from the hospital, some were still suffering from the same mental distress that they had experienced during the treatment duration.

Being in a higher fertility age group, having no child, and getting impaired fertility due to EP surgical treatment coupled with a series of deterring experiences, paved the way that women could hardly be able to adjust to their lives again. These deterring experiences, we named constant struggle, included *‘The well of loneliness’* and *‘Religion, a false savior.’*

***The well of loneliness:* S**ome married women considered EP a misfortune and impossible to tolerate. Although they needed someone to talk to, they found no one who could understand them truly. It seemed that they were trapped inside a well of loneliness. Apart from that, listening to people around, derived from the construction of the Sharia-driven cultural stereotypes in connection with women who cannot give birth, would make their pain and suffering double. Regardless of how strong they tried to be, this situation prevented them from accepting what had happened. A young woman who experienced this kind of loneliness stated:

My sister-in-law had come to the hospital, and ridiculously said: ‘You are an inferior and stupid person who couldn’t keep up your pregnancy,’ Following that my husband said, ‘You were weak. It was obvious that your ovaries were weak’. I got so upset to hear that (Zahra, 15 years old).

Likewise, another woman said:

I couldn’t get pregnant for 9 years, so [I was] rejected by my husband's family. When I got a positive pregnancy result, I would like to go there and say mission accomplished happily, but it was EP and another misery again (Najme, 35 years old).

As a woman put it:

I have a boy from my first marriage, but my current husband doesn’t. He wants his biological child, otherwise, I will lose my marital life (Fatemeh, 30 years old).

***Religion, a false savior:*** Due to the dominance of Islamic Sharia-driven in Iran, religion is the most available knowledge/belief system which could account for and answer several questions effortlessly. Regarding religious education in Iranian school textbooks, training requirements and principles of religion have always been accompanied by the fear of eternal punishment in Hell.

To shed more light, girls learn that failure to keep their Hijab in the public space or the presence of any male out of their family members will cause them to face Allah’s wrath on judgment day and burn in Hell. Then, they will be aware that the *Hijab* is for their safety and security in society. Consequently, since individuals have learnt about Hell before Heaven, they may consider bitter events, such as an EP, as a punishment for the sin that they probably have committed in life. As a woman said:

I assume it must be compensation for my guilt. Since I always felt guilty because of my intentional abortion I had before. I think as I did this guilt deliberately, I'd compensate for it one day. It was the first thing stuck in my mind (Tara, 20 years old).

From the religious perspective, discomfort in life always seems to be a test or punishment, and the participants who always felt themselves in a state of punishment, not only could not be productive in the community, but also remained in “the Hell” due to sociocultural stereotypes. So, faith ended, and mental illnesses overwhelmed the whole of their lives. A young woman who was plagued in her negative religious thoughts declared:

My mother-in-law was opposed to our marriage, so she expelled my husband from the family after we got married. Now I think maybe I caused a mother and son to stand apart from each other. His mother cursed me, and this happened (Fetemeh, 34 years old).

### Renascence in Life

Renascence in life refers to the contributing factors that assisted the women to cope with all challenges linked to EP and readapt to their lives regardless of the consequences. According to the women’s narratives, there were two influential contributors: demographic characteristics and social determinants. The primary findings of the research suggested that age, number of children, and the treatment results led to different experiences.

In other words, each woman who belonged to a lower fertility age group had at least one child and who passed through the treatment process with no complications, could tolerate the event. These experiences accompanied by three themes that stemmed from the interviews were deemed as some facilitators to readapt to life: *‘Human capital,’* *‘Faith as the key of heaven,’* and *‘Cosmic mysticism.’*

***Human capital:*** Human capital consists of two human assets: social support and cultural capital. Social support refers to a set of potential resources, depending on the ownership of a sustainable network of more or less institutionalized relationships.

To be more precise, it is a natural human primary need to have a supportive person to share their emotions and help reduce stress. From this viewpoint, the most prominent person to release a woman from illness is her husband. As a woman explained:

Regardless of what people would say, this belief that having your wife safe and sound, even with impaired fertility, is the most marvelous thing you have ever had, these were the words of my husband [which] could help me smile again (Huriya, 40 years old).

On the other hand, growing up in families that are used to teaching their girls independence and willpower in decision-making made them able to look at phenomena as if they were just different parts of a typical life. In addition, cognitive ability to consume cultural goods and higher education, all consisted of cultural capital—the exact thing that made some women able to come back to their routine lives. As a woman remarked:

I thought it was a disease truly like the others. Why did it happen? I didn’t know. My father always told me that life is full of ups and downs, no matter who are you, woman or man, and everyone is strong enough to tackle issues. I trusted doctors, believing [they] knew what to do. It probably had some reasons but wasn’t incurable (Elmira, 30 years old).

***Faith as the key to heaven:**** What happened to me,* and *who was liable for?* These were two key questions that Iranian women with EP keep asking themselves these questions and answered by spiritual faith.

In the Islamic beliefs, which primarily inherited in Iranian families, no events are catastrophic since God is a spiritual, superhuman, and invisible entity whose authority is always in turn to people's benefit. God's expediency means God has a redemptive purpose for everything that happens to his believers. As a result of this faith, Iranian women were abled to endure every difficult situation. As a believing woman expressed:

God examines our patience! It was definitely about God's expediency. God never puts me on the wrong track. If it was inside the uterus if it was born and then would died… or maybe it might have been defective or would be disabled. Surely it was the wisdom of God (Fatemeh, 19 years old).

And a woman detailed:

Yes, it was awful. But now, when I think about those days, I can see there were ten of us [women with EP] hospitalized in a room. I think it was God-gifted to find such good friends with mutual sense and common pain (Maryam, 34 years old).

***Cosmic mysticism*:** Since some women had lost their hopes and felt like falling to the bottom of a valley, something inside reminded them about their children who were alive, compared themselves with others in worse conditions, and started to readapt to life. A woman explained:

Today, I love living longer. The past is past. I found my spouse as worthy, my little girl a value, my health as a great asset, and a number of things to be proud of. Life still has something worth living (Reyhaneh, 40 years old).

Those women who support this view, which is in line with Islamic thought, argue that most diseases have a psychological basis. Therefore, it could easily be healed by a firm relationship with mysticism, love, and optimism. One woman declared:

Thanks to life, I was strong enough to find my power which was instilled into me by Mother Nature. I do believe that I am the supreme creature, the universe always watches over me. Mysticism and love can always help, even to do a miracle (Morvarid, 34 years old).

**Fig. 1:**
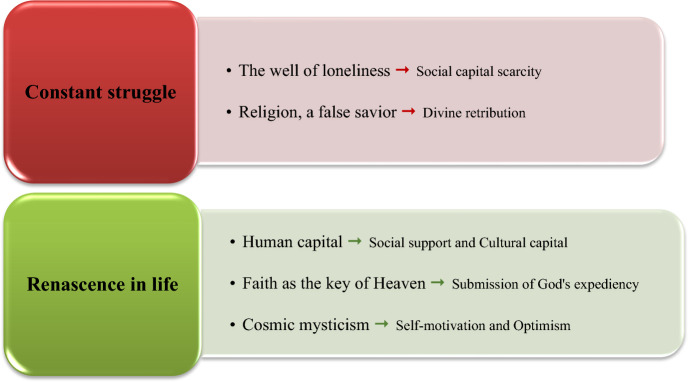
Themes and subthemes of the study

An overview of the main themes, themes, and subthemes extracted from the deep descriptions and interpretations of the interviews is presented in Fig. 1.

## Discussion

This study aimed to explore the perception and lived experience of married women with EP for the first time in the Islamic Republic of Iran. The findings of the study indicated that having a child or becoming a mother is unquestionably a significant part of women's identity in the pronatalist societies in the Middle East, including the Islamic Republic of Iran.

Moreover, EP as the same with every type of early pregnancy loss can be supposed as a traumatic event (Krosch & Shakespeare-Finch, 2017) that might persist for a long period of time (Farren & Jalmbrant, 2016).

It has been found that women do not experience traumatic situations equally. Some women have access to social and environmental support, which leads to a more positive experience that we call a "renascence in life." However, other women suffer greatly from harsh religious beliefs and sociocultural stereotypes that limit them to a "constant struggle."

In terms of the facilitators, human capital, faith, and cosmic mysticism play the key roles of ‘renascence in life’ which allows the women to accept the situation and continue the previous routine life.

While individual self-care tendency was significant among various patients (Fairweather et al., 2017), we found that social support provided these women with much more benefits. The study revealed that the husband plays a vital role in caring for women during the treatment of EP, which has a significant positive impact on their long-term satisfaction. (Rose, 2015).

In other words, while all women have lived in the shadow of Islamic Sharia law, only those participants who enjoyed the emotional and social support of their husbands have been less suffered from social oppression and stereotypes.

This study also illustrates how forms of capital, including cultural capital, can influence Iranian women's meanings and experiences of EP. As Pierre Bourdieu elucidated, cultural capital points to the collection of symbolic elements, such as skills, tastes, posture, clothing, mannerisms, material belongings, and credentials. According to Bourdieu (1986), cultural capital comes in three forms: embodied, objectified, and institutionalized.

In terms of the institutionalized form, cultural capital refers to credentials and qualifications such as degrees or titles that symbolize cultural competence and authority (Huang, 2019). In this view, having information and communication literacy, higher education, and social awareness could make an assertive cultural capital and confidence for Iranian women to manage and overcome all sociocultural oppressions and facilitate their adaptation to the rest of their lives.

Since Bourdieu points out that certain forms of cultural capital are valued over others which can help or hinder one’s social mobility just as much as income or wealth (Bourdieu, 1986), we could argue cultural capital brought about different experiences to the participants. Mackenbach (2012), Kamin et al. (2013), and Pinxten and Lievens (2014) considered cultural capital as a promising approach to explaining health inequalities and promoting health, happiness, and satisfaction.

As for faith as the key to Heaven, some Iranian women with EP experience confirmed that spiritual beliefs could support their mental health. In Iran, most people are used to ascribing the cause of every strange and inexplicable event to God/Allah. Those women who are in favor of this perspective argue that from their childhood they have been taught God always leads humans to the path of goodness, so everything that happens is due to God's expediency. It stems from the spiritual training and socialization ways of Iranian families, not the Islamic government or the society as a whole.

As several earlier studies (Lucchetti et al., 2012; Sankhe et al., 2017) in the literature revealed, the treatment of illnesses particularly mental illnesses has been integrated with spiritual therapy and has been accepted by patients and their family members (Lucchetti et al., 2012). Likewise, Sankhe et al. (2017) confirmed that faith in the treatment process strengthens people, helps them alleviate stress, and promotes patients to achieve a higher level of health regardless of external social pressures.

In line with Islamic thought, ‘cosmic mysticism’ might be deemed as a new wave of optimism in Iranian society which has been considered in recent decades. According to the findings, one thing that releases patients from mental distress is mysticism, which encourages believers to pay attention to the bright side of everything.

Hence, it could assist the patients to deal with every consequence and return to their typical daily lives. As some studies confirmed, this person-centered approach can be applied in a practical way to the theory and practice of psychotherapy (Thorne, 2002).

On the other hand, ‘constant struggle’ consisted of two themes: ‘The well of loneliness’ and ‘Religion, a false savior.’

In addition to the fact that childbirth is a necessity for families in the existing Islamic Sharia-driven Iran, having a grandchild from their son, particularly a grandson, who preserves the last name and originality of the husband's family, is of specific significance. All interviewed women believed that they had experienced dreadful loneliness from their spouses’ families. They stated that they needed someone to listen to them while they were blamed by the individuals who they did not expect.

Similar research on infertility and women’s illnesses suggested that living in an extended family in Iran, due to inappropriate family intrusions, can have an unpleasant impact on women’s life satisfaction, posing psychological pressure during assisted reproduction (Bakhtiyar et al., 2019). The findings of this study indicated a dysfunctional form of social capital which along with scarcity of social support could serve the participants to remain with mental disorders.

Moreover, the dominance of the Islamic Sharia-driven rules in post-revolutionary Iran has constructed and reconstructed the view that every traumatic and unpleasant event in life is a divine exam or punishment from God as a result of human wrongdoing. Having a similar view, some religious people in contemporary Iran consider events or disasters such as earthquakes, floods, and drought as a result of increasing sin in the society.

Regarding the prevalence of such a belief, it should be argued that in a society where religious education prioritizes a system of punishment over reward, there is no doubt that believers evaluate and interpret the downs of life as the measures of sin.

As a recent study revealed, Iran has a death-conscious culture, and the existence of this culture, which is manifested through the religious teachings, plays a role in the experiences of depression (Mirdamadi, 2019). Consequently, EP for women living in this context means a sign of divine retribution for a sin committed even if individuals are not aware of it.

In other words, the rigid pieces of training of Islamic Sharia law that cause scrutiny of all acts of the women based on trial and punishment leave no way to resuscitate individuals and fill them with despair of the future. One reason for acceptance of this worldview in Iranian families is ‘fatalism’ which imposed the women a compulsory acceptance of EP as a part of their destiny. Although these sorts of religious beliefs might alleviate the circumstance temporarily, they could turn the women's lives into living in such a long-term Hell.

Another reason refers to education and the amount of scientific knowledge. To be more precise, the educational level Iranian women have, the more priority they give to religious beliefs in order to perceive the event. In the shadow of Islamic Sharia culture and law, when there is a religious interpretation of the events, scientists are not allowed to hesitate in understanding, accepting, and applying that interpretation.

This study pointed that many women especially those who were in older ages with no children or recurrent miscarriage experience, neither were able to accept the therapeutic consequences, nor to provide a new definition of themselves in accordance with the new conditions. In line with the results of a recent study (He, 2019), because of losing one fallopian tube and reduction of reproductive function, these participants experienced a wide range of negative emotions including anxiety, anger, and depression more than the others.

### Study Limitations

Under the existing interpretation of Islamic Sharia law enforced since the 1979 Islamic revolution in Iran, any intimate relation that takes place between men and women, without or outside of marriage, is illegal (Rodziewicz, 2020). According to the Quran, extra-marital sex is one of the greatest sins and has a severe punishment (Quran, 60: 12; 25: 68; 17: 32).

In such cases, where the offenders do not meet the conditions of *Ihsan*, married to someone else, the *hadd* punishment for *zina*, illicit sex or adultery, shall be one hundred lashes (Islamic Penal Code, Article 230). Also, an illegitimate child or *valadalzina* does not receive inheritance from the father, the mother, or their relations (Civil Code of the Islamic Republic of Iran, Article 884).This is coupled with the fact that if unmarried women with EP should be referred to the healthcare center, culturally they should not discuss their emotions and feelings frankly, since they might be stigmatized as a prostitute.

For these reasons, this study focused merely on formally married women, the permanent or civil marriage, and other groups of women, for example, those women who cohabit with their partners under an arrangement known as a white marriage and have made a painful decision to get pregnant and unmarried in such a context with similar sexual and reproductive problems were not included in the study.

Therefore, it is recommended to design and carry out similar qualitative studies on women who experience EP with temporary or *sigheh* marriages and other forms of cohabitation in the Islamic Republic of Iran and other Islamic communities around the world. According to the Islamic Sharia-driven rules, there are two recognized types of marriage: *nekah*, formal marriage, and *sigheh*, temporary or in Arabic *mut'a* marriage (Civil Code of the Islamic Republic of Iran 1928; Iran: Islamic Penal Code, 1991; Khawaja and Mowafi 2006).

A *sigheh* can last from one hour to 99 years. The couple can sever this relationship without a legal divorce according to Iranian Civil Law Article 1113. A contract for a *sigheh* does not make the man responsible for financially supporting the woman. She has no right to inheritance or similar rights that women have in a permanent civil marriage.

While in the Shia Islam the later type has been legal since the time of the Islamic Prophet Mohammad (Hasannia & Masoudian, 2021), it is socioculturally stigmatized and viewed as a taboo in the public sphere. Most *sigheh* women suffer from vulnerability and insecurity, and most men keep their *sigheh* relationship secret (Yaghoobi, 2020). Deep consideration and comprehension of the meanings and experiences of *sigheh* women with health problems and reproductive losses such as EP by healthcare providers and policymakers are recommended.

Furthermore, it should be considered that these accounts and narratives were obtained from married women purposefully recruited from a specific area of North Iran and could not be generalized to other married or unmarried women with experience of EP around the multicultural and transitional society of Iran.

## Conclusion

This phenomenological study shed light on the point that how hegemony of a top-down, authoritative, and pronatalist Islamic Sharia-driven discourse in post-revolutionary Iran has greatly influenced cultural meanings, perceptions, memories, imaginations and lived experiences of married women with EP in everyday life and turned EP into a multidimensional pervasive trauma for them.

These women need to have social and psychological support to know that they have not lost their dignity, agency and identity, and life is still going on. They should be supported through resources developed in an understandable way, accompanying the management and removal of social oppressions including gender stereotypes constructed and reconstructed by religious extremists in Iranian society and government.

Hence, social support, effective communication, and positive interactions particularly from the significant others (e.g. their spouse, relatives and friends) and medical staff and healthcare providers could empower these married women to recover from their reproductive loss and prevent prolonged distress and social suffering.
